# Association Between Waist‐to‐Height Ratio Estimated Fat Mass Categories and Incident Fractures

**DOI:** 10.1002/jcsm.13834

**Published:** 2025-06-05

**Authors:** Yongin Cho, Jong Hyun Jhee, Namki Hong, Hye‐Sun Park

**Affiliations:** ^1^ Department of Endocrinology and Metabolism Inha University School of Medicine Incheon Republic of Korea; ^2^ Division of Nephrology, Department of Internal Medicine, Gangnam Severance Hospital Yonsei University College of Medicine Seoul Republic of Korea; ^3^ Department of Internal Medicine, Severance Hospital, Endocrine Research Institute Yonsei University College of Medicine Seoul Republic of Korea; ^4^ Division of Endocrinology, Department of Internal Medicine, Gangnam Severance Hospital Yonsei University College of Medicine Seoul Republic of Korea

**Keywords:** body mass index, fracture, obesity, osteoporosis, waist‐to‐height ratio

## Abstract

**Background:**

Although obesity is a well‐known risk factor for various metabolic disorders, its impact on fracture risk remains uncertain. The inconsistency in findings may be due to the fact that most studies have relied solely on body mass index (BMI) as the measure of obesity. Other indices, such as waist circumference (WC) and the waist‐to‐height ratio (WHtR), have been suggested as better indicators of obesity. This study aimed to evaluate the association between obesity and fracture risk by using multiple obesity measures, including WHtR, WC and BMI, in a longitudinal cohort.

**Methods:**

In this prospective cohort study, we analysed data from 5905 participants in the Korean Genome and Epidemiology Study (KoGES), with a median follow‐up of 16 years. Participants were categorized into tertiles based on WHtR, WC and BMI, and the incidence of overall fractures and site‐specific fractures (vertebral, hip and wrist/humerus) was assessed. Multivariate Cox proportional hazards models were used to examine the association between WHtR, WC, BMI and fracture risk, adjusting for potential confounders.

**Results:**

Among 5905 participants (54% women; age range, 40–69; median age, 50 years; interquartile range, 44–59), 816 fractures were reported over a median follow‐up period of 16 years. A one‐standard deviation increase in WHtR was associated with a 55% higher risk of overall fractures (adjusted hazard ratio [aHR] 1.55, 95% confidence interval [CI] 1.37–1.75), with similar trends observed for vertebral (aHR 1.60, 95% CI 1.13–2.26), hip (aHR 1.85, 95% CI 1.40–2.43) and wrist/humerus fractures (aHR 1.42, 95% CI 1.16–1.74). A one‐unit increase in WC was linked to a 16% higher risk of overall fractures (aHR 1.16, 95% CI 1.08–1.24). BMI was not significantly associated with the fracture risk. Within the same obesity group defined by BMI, participants in the higher WHtR tertiles had a greater incidence of overall fractures. Specifically, individuals in the third tertile of WHtR with a BMI of ≥ 23 to < 25 kg/m^2^ or ≥ 25 kg/m^2^ had a higher risk of overall fractures compared to those in the first tertile of WHtR with a BMI < 23 kg/m^2^ (aHR 1.88, 95% CI 1.34–2.62, and aHR 1.93, 95% CI 1.30–2.87, respectively).

**Conclusion:**

Although a high BMI has often been considered a protective factor against fractures, this study found that obesity, as measured by WHtR, is a risk factor. Even among individuals with a high BMI, those with elevated WHtR should receive additional medical attention to help prevent fractures.

## 1 Introduction

Fractures, the most severe consequence of osteoporosis, have profound implications, including deformity, disability, loss of independence, diminished quality of life and increased mortality, morbidity and healthcare costs [1]. Consequently, screening patients at high risk of fracture is crucial, and extensive efforts have been made to identify the associated risk factors. Obesity is one of the main risk factors for various adverse health outcomes, including metabolic disorders, such as diabetes, hypertension (HTN), cardiovascular disease (CVD) and increased mortality [2]. However, the relationship between obesity and fractures is complex. While a low body mass index (BMI) is a well‐known risk factor for fractures, a high BMI is generally presumed to offer protection against fractures [3]. Recent studies, however, have yielded mixed results regarding individuals with a high BMI. A study involving postmenopausal women found that increased BMI was associated with a higher risk of humerus fractures but a lower risk of hip fractures [4]. Similarly, Pirro et al. reported a positive association between BMI and an increased risk of vertebral fracture in postmenopausal women with osteoporosis [5]. These results raise concerns that, contrary to traditional beliefs, obesity may not be protective against fractures.

One possible explanation for these inconsistent results is the widespread use of BMI as the primary measure of obesity. While BMI is the most commonly used parameter for assessing obesity, its effectiveness as an accurate measure of obesity has been questioned because it does not differentiate between lean and fat mass [6]. To address the limitations of traditional anthropometric indicators, waist circumference (WC) and waist‐to‐height ratio (WHtR) were suggested as a better index for obesity‐related health risk [7]. The use of WHtR as a marker of abdominal obesity was first proposed in the mid‐1990s, and evidence has since accumulated [8]. A number of studies have associated WHtR not only with incident chronic kidney disease, HTN and diabetes but also with myocardial infarction and cardiovascular mortality [9, 10].

This study aimed to assess fracture risk in an obese population using WHtR, WC and BMI as measures of obesity, based on data from the Korean Genome and Epidemiology Study (KoGES) cohort with longitudinal follow‐up.

## 2 Methods

### 2.1 Study Population

Participants in this study were enrolled from the KoGES Ansan‐Ansung cohort, a prospective community‐based cohort established to investigate the genetic and environmental factors contributing to prevalent chronic diseases in South Korea [11]. The cohort, initiated in 2001, consists of 10 030 individuals aged 40–69 years from the rural area of Ansung and the medium‐sized city of Ansan, located near Seoul. The participants underwent initial medical evaluations, anthropometric measurements and blood sampling, followed by biennial health examinations and surveys from 2001 to 2020.

Among the 10 030 individuals initially enrolled, we excluded those with missing WC, body weight or height data at baseline (*n* = 13), who were diagnosed with cancer and receiving treatment (*n* = 15), who were treated under the diagnosis of cancer (*n* = 28), individuals with a BMI less than 18.5 kg/m^2^ (*n* = 188) and those who did not respond to the fracture questionnaire during the follow‐up period (*n* = 3881). After these exclusions, 5905 individuals were included in the study. All the participants voluntarily participated in the study and provided informed consent. This study was conducted in accordance with the Declaration of Helsinki and approved by the Ethics Committee of KoGES at the Korean National Institute of Health and the Institutional Review Board of the Yonsei University Health System Clinical Trial Centre (32024‐0297).

### 2.2 Data Collection

Anthropometric measurements, including body height, weight and WC, were taken at baseline by trained research staff while the participants were fasting and wearing light clothing. WC was measured to the nearest 0.1 cm at the midpoint between the lower margin of the last rib and the crest of the ilium using a horizontal plane. BMI was calculated by dividing the weight in kilograms by the height in meters squared (kg/m^2^). Blood samples were collected after an 8‐h fast and transported within 24 h to a central laboratory (Seoul Clinical Laboratories, Seoul, Republic of Korea) for analysis. Data on CRP levels were subsequently obtained.

Baseline clinical data, including alcohol consumption, smoking history, income level, physical activity and histories of HTN and diabetes, were collected using self‐report questionnaires. Participants' income levels were categorized into tertiles based on the average monthly income per person: low (< $750/month), middle ($750–$1500/month) and high (≥ $1501/month). Physical activity was defined as ‘active’ if participants engaged in at least 150 min of moderate exercise per week, and ‘inactive’ if they did not meet this criterion. The bone ultrasonic speed of sound (SoS, m/s) was measured at baseline using quantitative ultrasonography (QUS, Omnisense 7000 s, Sunlight Medical Ltd., Petah Tikva, Israel). The measurement was taken at the midpoint between the patella and medial malleolus of the less frequently used leg. The final SoS value for the midshaft tibia was determined by averaging the results of three consecutive measurements.

### 2.3 Exposure

We designated WHtR as the main exposure of interest in this study because traditional obesity indices, such as BMI, have produced inconsistent results regarding fracture outcomes. To calculate WHtR, we divided WC (cm) by height circumference (cm). WHtR was analysed as both a continuous and a categorical variable. For categorical analysis, the participants were divided into three groups based on the tertiles of WHtR, which were calculated separately for each sex. For women, those with a WHtR ranging from 0.368 to 0.500 were categorized as Group 1, from 0.501 to 0.561 as Group 2 and from 0.562 to 0.813 as Group 3. For men, the WHtR ranges were 0.380–0.483 for Group 1, 0.484–0.522 for Group 2 and 0.523–0.671 for Group 3.

### 2.4 Outcome

The primary endpoint was the first occurrence of a fracture. During the follow‐up period, participants completed a biennial self‐reported questionnaire to document any new fracture events, including the age at which the fracture occurred, and the specific sites involved. The overall fracture category included any fractures reported by the participants, except those resulting from accidents or falls from heights greater than standing height, as well as fractures of the fingers and toes. Vertebral, hip and wrist/humerus fractures were separately examined for the analysis of site‐specific fractures. The primary analysis focused on the first occurrence of a fracture and its associated site.

### 2.5 Secondary Analysis

Several secondary analyses were conducted to confirm the robustness of the findings. First, the risk of fracture was analysed separately in women and men because the effect size of WHtR might differ depending on sex, and fracture events usually affect women. Second, BMI and WC were evaluated as exposure variables because these two parameters are commonly used as traditional anthropometric measurements to assess obesity. In the categorical analysis, participants were grouped into tertiles of BMI and WC, which were calculated separately for each sex. For women, the BMI groups were 18.5–23.4, 23.5–25.9 and 26.0–40.2 kg/m^2^ for Groups 1, 2 and 3, respectively. For men, BMI was categorized as 18.5–23.0, 23.1–25.6 and 25.7–35.9 kg/m^2^ for Groups 1, 2 and 3, respectively. Similarly, WC was categorized into three groups for women: 56.0–76.9, 77.0–85.9 and 86.0–122.0 cm for Groups 1, 2 and 3, respectively. For men, the WC groups were 63.5–80.9, 81.0–86.9 and 87.0–112.0 cm for Groups 1, 2 and 3. Additionally, we categorized the study participants according to the World Health Organization obesity criteria. Participants were classified as obese if they had a BMI of ≥ 25 kg/m^2^, overweight if they had a BMI between 23 and 25 kg/m^2^ and normal or underweight if they had a BMI of < 23 kg/m^2^ [12]. Third, the association between WHtR and fracture was examined separately for individuals aged < 50 and ≥ 50. Lastly, WHtR cut‐off values were employed to ensure the consistency of the findings. According to previous reports, a WHtR of < 0.40 was classified as low body fat for both men and women. Normal body fat was defined as 0.40 ≤ WHtR < 0.50 in men and 0.40 ≤ WHtR < 0.51 in women. High body fat was categorized as 0.50 ≤ WHtR < 0.53 in men and 0.51 ≤ WHtR < 0.54 in women, while excessive body fat was defined as WHtR ≥ 0.53 in men and WHtR ≥ 0.54 in women [13, 14].

### 2.6 Statistical Analysis

For baseline characteristics, continuous variables are presented as medians with interquartile ranges (IQR) based on normality tests, including both Q‐Q plots and the Shapiro–Wilk test. Means ± standard deviations (SD) are also provided to better illustrate the data distribution. Categorical variables are reported as absolute numbers with corresponding percentages. Intergroup comparisons were conducted using the Kruskal–Wallis test for non‐normally distributed continuous variables, while categorical variables were analysed using chi‐square tests. Kaplan–Meier analysis with a log‐rank test was used to compare the cumulative incidence of fractures across the WHtR tertiles. Survival time was defined as the period from the baseline visit to the occurrence of the study outcome or last follow‐up. Multivariable Cox proportional hazards models were constructed to evaluate the associations between obesity indices and fracture risk. The covariates included in these models were age, sex, BMI, alcohol/smoking status, income level, history of HTN, diabetes or thyroid disease, SoS at the midshaft tibia, physical activity and CRP levels. The results were presented as adjusted hazard ratios (aHR) with 95% confidence intervals (CI). To illustrate the fracture incidence across WHtR tertile groups and obesity groups, a bar graph was generated. In this analysis, a Poisson regression model was used to assess fracture incidence across the groups, with normal weight and WHtR Group 1 designated as the reference group. The significance of the differences in fracture rates between groups was tested based on the estimated coefficients and *p*‐values derived from the model. To illustrate the relationship between incident fracture risk and WHtR, BMI and WC as continuous variables, restricted cubic spline analysis was performed, adjusting for age and sex. Extreme outliers, defined as values less than the first quartile (Q1) minus 1.5 times the IQR or greater than the third quartile (Q3) plus 1.5 times the IQR, were excluded from the cubic spline analysis. To illustrate the association between WHtR and SoS at the midshaft of the tibia, scatter plots were generated, and Pearson's correlation test was applied. Statistical significance was set at *p* < 0.05. All statistical analyses were conducted using R software version 4.3.2.

## 3 Results

### 3.1 Clinical Characteristics

A total of 5905 participants (median age 50.0 [IQR 44.0–59.0] years, 54% women) were enrolled in the study. The participants were divided into three groups based on their WHtR tertiles (Table 1). Individuals in the higher WHtR tertile group tended to be older and had higher BMI, WC and CRP levels. They also had lower SoS at the midshaft tibia. Additionally, a higher proportion of individuals in the higher WHtR tertile group had a greater prevalence of HTN and diabetes and lower income levels.

**TABLE 1:** Baseline characteristics of the patients according to WHtR tertiles.

		Group 1	Group 2	Group 3	*p*‐value
		*N* = 1969	*N* = 1968	*N* = 1968	
WHtR range	Men	0.380–0.483	0.484–0.522	0.523–0.671	
	Women	0.368–0.500	0.501–0.561	0.562–0.813	
Age, years		46 [43–53]; 49 ± 8	50 [44–58] 51 ± 8	55 [47–62] 55 ± 8	< 0.001
Women, n (%)		1065 (54%)	1064 (54%)	1064 (54%)	> 0.9
BMI, kg/m^2^		22.2 [20.9–23.6]; 22.3 ± 1.9	24.7 [23.3–26.1]; 24.7 ± 2.1	26.9 [25.5–28.7]; 27.2 ± 2.6	< 0.001
Weight, kg		58 [53–63]; 58 ± 8	63 [57–70]; 63 ± 9	68 [61–75]; 68 ± 10	< 0.001
Height, cm		161 [155–168]; 162 ± 8	159 [153–167]; 160 ± 9	158 [151–165]; 158 ± 9	< 0.001
Waist circumference, cm		75 [71–77]; 4 ± 5	83 [80–85]; 83 ± 4	91 [88–95]; 92 ± 5	< 0.001
Alcohol drink, current, n (%)		958 (49%)	938 (48%)	870 (44%)	< 0.001
Smoking					0.4
Current, n (%)		513 (26%)	467 (24%)	444 (23%)	
Ex‐smoker, n (%)		269 (14%)	311 (16%)	331 (17%)	
Never, n (%)		1187 (60%)	1190 (60%)	1193 (61%)	
Physical activity, active, n (%)		1105 (58%)	1194 (63%)	1219 (65%)	< 0.001
Hypertension, n (%)		122 (6.2%)	273 (14%)	476 (24%)	< 0.001
Diabetes, n (%)		55 (2.8%)	114 (5.8%)	178 (9.0%)	< 0.001
Thyroid disease, n (%)		23 (1.2%)	15 (0.8%)	20 (1.0%)	0.4
Income					< 0.001
High		917 (47%)	747 (38%)	539 (27%)	
Middle		614 (31%)	577 (29%)	546 (28%)	
Low		438 (22%)	644 (33%)	883 (45%)	
SoS at midshaft tibia, m/s		3966 [3877–4048]; 3958 ± 136	3919 [3811–4012]; 3908 ± 161	3885 [3760–3984]; 3870 ± 169	< 0.001
CRP, mg/dL		0.11 [0.05–0.19]; 0.19 ± 0.51	0.14 [0.07–0.23]; 0.23 ± 0.76	0.17 [0.08–0.29]; 0.26 ± 0.41	< 0.001

*Note:* Continuous variables are presented as median [interquartile range] and mean ± standard deviation. Categorical variables are presented as numbers (%).

Abbreviations: BMI, body mass index; CRP, c‐reactive protein; SoS, speed of sound; WHtR, waist‐to‐height ratio.

### 3.2 The Association Between WHtR and Fracture

**FIGURE 1:**
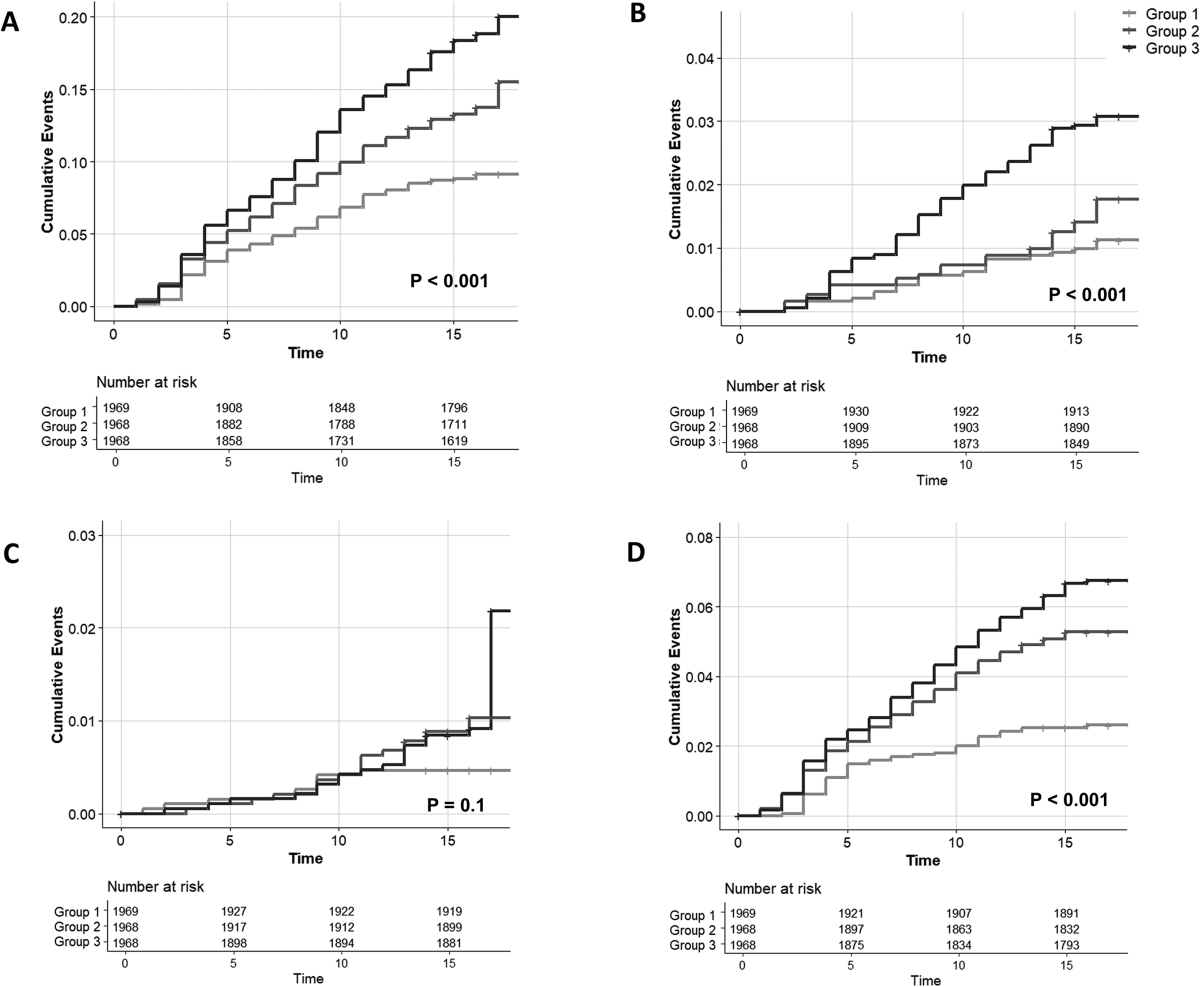
Kaplan–Meier curves for incident fracture incidence among WHtR tertile groups (A: overall fracture, B: vertebral fracture, C: hip fracture and D: wrist or humerus fracture).

During the median follow‐up of 16.0 [IQR, 15.0–16.0] years, 816 cases of overall fracture were observed (Table 2). The incidence rates of overall fractures were 6.03, 9.35 and 13.11 cases per 1000 person‐years in Groups 1, 2 and 3, respectively. A similar trend was observed across specific fracture sites, including vertebral and wrist/humerus fractures, with incidence rates increasing from Group 1 to Group 3. Kaplan–Meier curves demonstrated that the cumulative incidence of overall fractures, as well as vertebral and wrist/humerus fractures, significantly differed among the WHtR tertile groups, with higher tertiles being associated with a higher incidence rate (log‐rank test *p* < 0.001) (Figure 1A,B,D). However, the cumulative incidence of hip fractures did not differ significantly among the groups, possibly due to the low number of events (Figure 1C).

**TABLE 2:** Association between WHtR and incident fractures.

	Events	Person‐years	Incidence rate^a^	Model 1		Model 2		Model 3		Model 4	
				HR (95% CI)	*p*	HR (95% CI)	*p*	HR (95% CI)	*p*	HR (95% CI)	*p*
Overall fracture											
Per 1 SD increase				1.37 (1.29–1.46)	< 0.001	1.59 (1.41–1.79)	< 0.001	1.60 (1.42–1.80)	< 0.001	1.55 (1.37–1.75)	< 0.001
Group 1	178	29 529	6.03	Ref		Ref		Ref		Ref	
Group 2	269	28 760	9.35	1.55 (1.28–1.88)	< 0.001	1.63 (1.32–2.00)	< 0.001	1.63 (1.32–2.00)	< 0.001	1.56 (1.27–1.93)	< 0.001
Group 3	369	28 157	13.11	2.17 (1.81–2.60)	< 0.001	2.38 (1.86–3.05)	< 0.001	2.38 (1.86–3.05)	< 0.001	2.27 (1.76–2.92)	< 0.001
Vertebral fractures											
Per 1 SD increase				1.59 (1.35–1.87)	< 0.001	1.70 (1.23–2.34)	0.001	1.62 (1.16–2.27)	0.004	1.60 (1.13–2.26)	0.008
Group 1	21	30 340	0.69	Ref		Ref		Ref		Ref	
Group 2	32	30 016	1.07	1.54 (0.89–2.67)	0.123	1.46 (0.80–2.64)	0.214	1.41 (0.78–2.56)	0.257	1.42 (0.78–2.59)	0.254
Group 3	58	29 695	1.95	2.82 (1.71–4.65)	< 0.001	2.63 (1.34–5.15)	0.005	2.37 (1.20–4.70)	0.013	2.40 (1.20–4.81)	0.014
Hip fractures											
Per 1 SD increase				1.40 (1.07–1.82)	0.013	2.00 (1.20–3.34)	0.008	2.01 (1.20–3.37)	0.008	1.85 (1.40–2.43)	< 0.001
Group 1	9	30 349	0.30	Ref		Ref		Ref		Ref	
Group 2	19	30 127	0.63	2.14 (0.97–4.72)	0.061	2.26 (0.94–5.41)	0.067	2.22 (0.92–5.31)	0.074	2.06 (1.13–3.77)	0.019
Group 3	18	29 904	0.60	2.02 (0.91–4.50)	0.085	2.32 (0.78–6.88)	0.128	2.30 (0.78–6.82)	0.133	1.91 (1.04–3.52)	0.037
Wrist or humerus											
Per 1 SD increase				1.54 (1.39–1.71)	< 0.001	1.48 (1.22–1.81)	< 0.001	1.48 (1.21–1.80)	< 0.001	1.42 (1.16–1.74)	0.001
Group 1	50	30 157	1.66	Ref		Ref		Ref		Ref	
Group 2	102	29 567	3.45	2.08 (1.48–2.91)	< 0.001	1.89 (1.31–2.73)	0.001	1.87 (1.30–2.71)	0.001	1.82 (1.25–2.64)	0.002
Group 3	129	29 211	4.42	2.66 (1.92–3.69)	< 0.001	2.24 (1.45–3.47)	< 0.001	2.23 (1.44–3.45)	< 0.001	2.09 (1.34–3.26)	0.001

^a^ Per 1000 person‐years.

*Note:* Model 1: unadjusted. Model 2: adjusted for age, sex and body mass index. Model 3: adjusted for Model 2: alcohol consumption, smoking, income, hypertension, diabetes and thyroid disease. Model 4: adjusted for Model 3, SoS at the midshaft tibia, physical activity and CRP level.

Abbreviations: CI, confidence interval; CRP, C‐reactive protein; HR, hazard ratio; SoS, speed of sound; WHtR, waist‐to‐height ratio.

In Cox proportional hazard model, the risk of overall fracture was significantly higher in individuals in Groups 2 and 3 compared to Group 1 after adjusting for age, sex, BMI, alcohol/smoking status, income level, presence of HTN, diabetes or thyroid disease, SoS at midshaft tibia, CRP and physical activity (aHR 1.56, 95% CI 1.27–1.93, and aHR 2.27, 95% CI 1.76–2.92, respectively) (Table 2). Additionally, each 1‐SD increase in WHtR was associated with a 55% increased risk of overall fractures (aHR 1.55, 95% CI 1.37–1.75). A similar trend was observed for vertebral, hip and wrist/humerus fractures. Specifically, a 1‐SD increase in WHtR was linked to a 60% increased risk of vertebral fractures (aHR 1.60, 95% CI 1.13–2.26), an 85% increased risk of hip fractures (aHR 1.85, 95% CI 1.40–2.43) and a 42% increased risk of wrist/humerus fractures (aHR 1.42, 95% CI 1.16–1.74). Likewise, individuals in the higher WHtR tertile groups had an elevated risk of vertebral and wrist/humerus fractures, although the increased risk of vertebral fractures in Group 2 was not statistically significant.

### 3.3 Secondary Analyses

**FIGURE 2:**
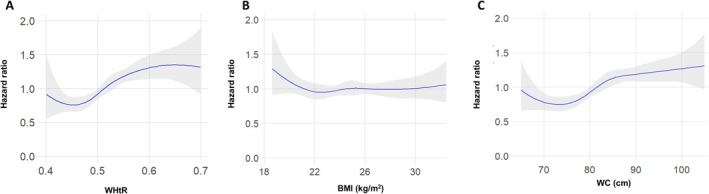
Cubic spline plot for incident overall fracture according to WHtR (A), BMI (B) and WC (C). *Note:* The blue line represents the hazard ratio, and the grey surface area represents the 95% confidence interval. The curve is adjusted for age and sex. Abbreviations: BMI, body mass index; WC, waist circumference; WHtR, waist‐to‐height ratio.

Several secondary analyses were conducted to confirm the robustness of the results. First, the analyses were performed separately according to sex (Table S1). Although the effect size was bigger in women, men with higher WHtR also had a significantly increased risk of fracture. The 1‐SD increase of WHtR in men was associated with a 49% increased risk of fracture (aHR 1.49, 95% CI 1.22–1.81). Moreover, men in Groups 2 or 3 had 1.45‐fold and 2.31‐fold increased fracture risk compared to those in Group 1 (aHR 1.45, 95% CI 1.05–1.99, and aHR 2.31, 95% CI 1.53–3.49, respectively). Secondly, we explored the relationship between BMI and WC, the traditional measures of obesity and fracture risk. Individuals in the highest BMI tertile tended to have a decreased risk of fracture compared with those in the lowest BMI tertile, but this was not statistically significant (aHR 0.91, 95% CI 0.76–1.09) (Table 3). When analysed by sex, only men in the highest BMI tertile had a significantly lower risk of fracture (aHR 0.74, 95% CI 0.56–0.98), while no significant association was found in women (aHR 1.03, 95% CI 0.82–1.29) (Table S2). A 1‐SD increase in BMI was not associated with fracture risk in the overall population or when men and women were analysed separately. The analysis of the association between WC and fracture risk showed a trend similar to that of WHtR. Individuals in the highest WC tertile had a 44% higher risk of fracture compared to those in the lowest WC tertile (aHR 1.44, 95% CI 1.20–1.72), but it was not significant among men (aHR 1.21, 95% CI 0.92–1.57). A 1‐SD increase in WC was linked to a 16% increase in fracture risk (aHR 1.16, 95% CI 1.08–1.24). However, this association was not observed in men (aHR 1.06, 95% CI 0.95–1.18). In the site‐specific analyses, neither BMI nor WC was associated with vertebral or hip fractures (Table 3). Only wrist/humerus fractures showed an association with a 1‐SD increase in WC (aHR 1.18, 95% CI 1.05–1.33), but not with BMI (aHR 1.05, 95% CI 0.93–1.18). Restricted cubic spline analyses indicated a gradual increase in the fracture risk with increasing WHtR and WC, as shown in Figure 2A,C. However, the analysis did not reveal a linear association between fracture risk and BMI (Figure 2B).

**TABLE 3:** Association between BMI, WC and incident fractures.

	BMI				WC			
	Unadjusted		Adjusted^a^		Unadjusted		Adjusted^a^	
	HR (95% CI)	*p*	HR (95% CI)	*p*	HR (95% CI)	*p*	HR (95% CI)	*p*
Overall fracture								
Per 1 SD increase	0.99 (0.92–1.06)	0.717	0.99 (0.93–1.07)	0.891	1.29 (1.20–1.38)	< 0.001	1.16 (1.08–1.24)	< 0.001
Group 1	1.00		1.00		1.00		1.00	
Group 2	0.99 (0.84–1.17)	0.902	1.03 (0.87–1.21)	0.761	1.35 (1.12–1.63)	0.001	1.18 (0.98–1.42)	0.082
Group 3	0.89 (0.75–1.06)	0.190	0.91 (0.76–1.09)	0.297	1.86 (1.57–2.22)	< 0.001	1.44 (1.20–1.72)	< 0.001
Vertebral fracture								
Per 1 SD increase	0.98 (0.81–1.18)	0.852	0.92 (0.76–1.13)	0.442	1.34 (1.12–1.61)	0.002	1.07 (0.88–1.30)	0.506
Group 1	1.00		1.00		1.00		1.00	
Group 2	1.31 (0.85–2.04)	0.224	1.40 (0.89–2.018)	0.141	1.15 (0.69–1.93)	0.594	0.90 (0.53–1.51)	0.680
Group 3	0.86 (0.53–1.39)	0.532	0.82 (0.49–1.38)	0.461	2.00 (1.26–3.18)	0.003	1.19 (0.73–1.95)	0.480
Hip fractures								
Per 1 SD increase	0.81 (0.59–1.09)	0.165	0.88 (0.65–1.19)	0.412	1.25 (0.94–1.65)	0.130	1.10 (0.82–1.47)	0.545
Group 1	1.00		1.00		1.00		1.00	
Group 2	1.05 (0.55–2.00)	0.879	1.21 (0.66–2.21)	0.541	1.81 (0.87–3.79)	0.113	1.52 (0.83–2.76)	0.172
Group 3	0.50 (0.22–1.10)	0.086	0.59 (0.28–1.22)	0.152	1.37 (0.63–2.99)	0.425	0.93 (0.49–1.76)	0.826
Wrist or humerus								
Per 1 SD increase	1.07 (0.95–1.20)	0.262	1.05 (0.93–1.18)	0.421	1.34 (1.19–1.50)	< 0.001	1.18 (1.05–1.33)	0.007
Group 1	1.00		1.00		1.00		1.00	
Group 2	0.90 (0.68–1.21)	0.495	0.93 (0.70–1.25)	0.634	1.57 (1.13–2.18)	0.008	1.27 (0.91–1.79)	0.159
Group 3	0.96 (0.72–1.27)	0.767	0.96 (0.72–1.29)	0.785	2.36 (1.73–3.21)	< 0.001	1.66 (1.21–2.30)	0.002

^a^ Adjusted for age, sex, alcohol consumption, smoking, income, hypertension, diabetes, thyroid disease, SoS at the midshaft tibia, physical activity and CRP level.

Abbreviations: BMI, body mass index; CI, confidence interval; CRP, C‐reactive protein; HR, hazard ratio; SoS, speed of sound; WC, waist circumference.

As obesity is typically classified by BMI, we assessed the fracture risk based on the BMI‐defined obesity groups and WHtR categories. Within obesity groups, individuals in the higher WHtR tertiles had a higher incidence of overall fractures (Figure S1). Among the obese group (BMI ≥ 25 kg/m^2^), those in WHtR Group 1 had the lowest fracture incidence rate, while individuals in WHtR Group 3 had a rate of 12.2 per 1000 person‐years. This rate was higher than that observed in normal weight individuals (BMI < 23 kg/m^2^) in WHtR Group 1 (aHR 1.93, 95% CI 1.30–2.87) (Figure S2).

To confirm the robustness of the association between WHtR and fractures, the analysis was stratified by age group (< 50 and ≥ 50) (Table S3). In both age groups, an increase of 1‐SD in the WHtR was associated with a higher fracture risk (aHR 1.59, 95% CI 1.30–1.95 for age < 50 and aHR 1.50, 95% CI 1.29–1.75 for age ≥ 50), with higher tertiles being associated with a greater incidence rate. Additionally, WHtR cut‐off values were employed to test the consistency of the results (Table 4). The distribution of individuals across body fat categories was as follows: very low body fat, *n* = 28; normal body fat, *n* = 2446; high body fat, *n* = 1247; and excessive body fat, *n* = 2184. Compared to the normal body fat group, both the high and excessive body fat groups exhibited an increased risk of fractures (aHR 1.55, 95% CI 1.25–1.92, and aHR 2.00, 95% CI 1.60–2.51, respectively) after adjusting for multiple covariates. In contrast, the fracture risk in the very low body fat group was not statistically significant. However, given that this group comprised only 28 participants, the analysis may have been underpowered to detect a meaningful association.

**TABLE 4:** Association between WHtR estimated fat categories and incident fractures.

Overall fracture	Events	Person‐years	Incidence rate^a^	Model 1		Model 2		Model 3		Model 4	
				HR (95% CI)	*p*	HR (95% CI)	*p*	HR (95% CI)	*p*	HR (95% CI)	*p*
**WHtR categories**											
Low fat	3	405	7.41	1.19 (0.38–3.72)	0.763	1.22 (0.39–3.83)	0.733	1.23 (0.39–3.87)	0.721	1.23 (0.39–3.86)	0.722
Normal fat	228	36 593	6.23	Ref		Ref		Ref		Ref	
High fat	178	18 209	9.78	1.57 (1.29–1.91)	< 0.001	1.61 (1.30–1.98)	< 0.001	1.61 (1.30–1.98)	< 0.001	1.55 (1.25–1.92)	< 0.001
Excess fat	407	31 239	13.03	2.09 (1.78–2.46)	< 0.001	2.10 (1.68–2.62)	< 0.001	2.10 (1.68–2.62)	< 0.001	2.00 (1.60–2.51)	< 0.001

^a^ per 1000 person‐years.

*Note:* WHtR categories—Men: < 0.40 (low fat), 0.40–0.49 (normal fat), 0.50–0.52 (high fat) and ≥ 0.53 (excessive fat); Women: < 0.40 (low fat), 0.40–0.50 (normal fat), 0.51–0.53 (high fat) and ≥ 0.54 (excessive fat). Model 1: unadjusted. Model 2: adjusted for age, sex and body mass index. Model 3: adjusted for Model 2: alcohol consumption, smoking, income, hypertension, diabetes and thyroid disease. Model 4: adjusted for Model 3, SoS at the midshaft tibia, physical activity and CRP level.

Abbreviations: CI, confidence interval; CRP, C‐reactive protein; HR, hazard ratio; SoS, speed of sound; WHtR, waist‐to‐height ratio.

To understand the mechanisms underlying the association between WHtR and fracture, we examined the correlation between WHtR and bone mineral density (BMD) measured using SoS at the midshaft tibia. Scatter plots showed a significant negative correlation between WHtR and BMD (*R* = −0.36, *p* < 0.001) (Figure S3).

## 4 Discussion

Using a large prospective cohort study, we found that obesity, as measured by WHtR, was associated with an increased risk of incident fractures. This relationship was consistent regardless of sex. However, BMI, a traditional indicator of obesity, did not show a consistent association with incident fractures. Notably, an association between high WHtR and increased risk of incident overall fractures was evident, even in individuals classified as overweight or obese based on BMI categories.

Although obesity is a well‐known risk factor for various metabolic diseases, its association with fracture risk is not fully understood. Underweight is a major risk factor for fractures; individuals with a BMI of less than 20 kg/m^2^ experienced a nearly twofold increase in the risk of hip fractures [3]. Conversely, obesity has traditionally been viewed as a protective factor against osteoporosis and fractures [15]. The association between obesity and reduced fracture risk is thought to be largely influenced by BMD, as a larger body size generates greater mechanical loading on the bones, leading to a higher BMD and lower fracture risk. A previous meta‐analysis revealed that individuals with a BMI greater than 25 kg/m^2^ had a 17% reduced risk of hip fractures (risk ratio 0.83, 95% CI 0.69–0.99) [3]. However, after adjusting for BMD, this association lost its significance, and there was no longer a linear relationship between BMI > 25 kg/m^2^ and fracture risk. Based on these findings, the authors suggested that obesity may not be a protective factor against fracture risk [3]. Several studies have reached similar conclusions. In a study by the Fracture Liaison Service involving 1005 women with low‐trauma fractures, 27.7% had a BMI > 30 kg/m^2^ [16]. Hsu et al. evaluated body fat mass using dual‐energy X‐ray absorptiometry and analysed its association with fracture risk in a Chinese cohort of 13 970 individuals. They observed that a high body fat mass was associated with an increased risk of nonspinal fractures [17]. Interestingly, they also showed that high fat mass was linked to an increased risk of osteoporosis, which contradicts previous beliefs and raises questions about the utility of BMI as a marker of obesity. With the rising prevalence of obesity, there is a pressing need for studies that accurately assess fracture risk in obese populations using more relevant obesity parameters.

Our study comprehensively analysed the relationship between fracture risk and obesity using WHtR, a marker for abdominal obesity. WHtR has been positively associated with HTN, diabetes, CVD and mortality—associations that are not consistently observed with other obesity measurements, such as BMI or WC [10, 18, 19]. Recently, the PARAGON‐HF trial demonstrated that central adiposity assessed by WHtR was significantly associated with adverse heart failure events [20]. Furthermore, a prospective birth cohort study in the UK reported that WHtR‐estimated excess fat was associated with a 6.08‐fold increased odds of developing type 2 diabetes [21].

Our findings revealed that individuals with a high WHtR had an increased risk of fractures. Specifically, a 1‐SD increase in WHtR was associated with a 55% higher risk of fracture (aHR 1.55, 95% CI 1.37–1.75), and those in the highest WHtR tertile had a 2.27‐fold higher risk of fractures compared to those in the lowest tertile (HR 2.27, 95% CI 1.76–2.92). Similarly, previous studies have reported that abdominal obesity, as measured by WHtR, is associated with a higher risk of vertebral or hip fractures [22, 23]. However, these studies were limited in that they either included only women or did not analyse fracture risk by specific site. In contrast, our study included both men and women and further validated these findings by examining fracture risk according to specific fracture sites.

In this prospective cohort study, WHtR was consistently associated with an increased risk of fractures, including vertebral, hip and wrist/humerus fractures. Interestingly, our study found no sex differences in the association between a high WHtR and increased fracture risk. Given that fat tissue can convert androgens to oestrogen, some authors have suggested that fat tissue in women may positively influence bone health [24, 25]. In contrast, men are generally less susceptible to fractures compared to women [26], making it more challenging to detect an increased fracture risk in studies. To consider these sex‐specific differences in bone health, we conducted a subgroup analysis based on sex. Our findings revealed a significantly increased risk of fractures in both men and women with a high WHtR. Notably, the presumed protective effect of adiposity on bone health in women was not observed, and instead, increased adiposity was associated with a higher fracture risk in both sexes.

As BMI is a traditional marker of obesity, we further analysed the association between BMI and fracture risk. While a high BMI has often been considered a protective factor against fractures, we did not find a solid link between BMI and fracture risk. Interestingly, obese individuals (BMI ≥ 25 kg/m^2^) with a high WHtR were found to have an increased risk of fractures. Typically, obese individuals are not considered a high‐risk group for fractures; however, this finding highlights the limitations of BMI in effectively identifying at‐risk individuals. Individuals with high WHtR should be recognized as a high‐risk group for fractures, even if they have a high BMI.

The mechanism by which WHtR is associated with an increased risk of fracture is not fully understood. One possible explanation for this finding is its relationship with BMD. In this study, individuals in the higher WHtR tertiles had lower BMD as measured by the SoS at the midshaft tibia. Similarly, a previous study found that BMD significantly decreased with increasing WHtR in women [27]. This finding may seem contradictory to those of previous studies that reported a higher BMD in individuals with a high BMI [28]. However, several studies have shown that excessive body fat mass or fat percentage can negatively affect BMD [17, 29, 30], as observed in our study. Several mechanisms have been proposed to explain the phenomenon. For instance, adipose tissue secretes proinflammatory cytokines including interleukin‐6 and tumour necrosis factor‐alpha, which can negatively impact bone mass [31, 32]. Additionally, obese individuals often have reduced levels of 25‐hydroxyvitamin D and elevated parathyroid hormone levels, both of which may accelerate bone resorption and weaken bone strength [15, 33]. Furthermore, several hormones produced by adipose tissue, including leptin, adiponectin and resistin, are dysregulated in obesity, further contributing to impaired bone health [34]. Nevertheless, we believe that other mechanisms underpin the association between WHtR and increased fracture risk, as this relationship remained robust even after adjusting for SoS values, warranting further investigation.

This study had several limitations. A major limitation of this study is the reliance on self‐reported questionnaires for fracture history, which could introduce recall bias. Some fractures may have been missed, particularly asymptomatic vertebral fractures. Additionally, bias could have been introduced by excluding participants with missing responses. However, our comparison with nationwide Korean data, which is based on medical records and diagnosis codes [35], suggests that the fracture incidence rate in our study is comparable to that of the general Korean population (data not shown). This is consistent with prior research showing that self‐reported fracture data generally show acceptable agreement with radiographic and medical records [36]. Second, this cohort consisted of individuals who were able to complete self‐administered questionnaires during biannual follow‐up visits, which may have introduced health volunteer bias. Additionally, the study lacked mortality data, meaning that participants lost to follow‐up due to death could not be accounted for, which may have influenced the findings. Third, BMD was assessed using QUS, which is not considered the gold standard for evaluating bone density; consequently, the analysis of the association between WHtR and BMD may be limited. However, previous studies have demonstrated that QUS‐measured BMD correlates well with DXA‐measured BMD and is a comparable predictor of fracture risk [37, 38]. Accordingly, QUS has been employed in large‐scale epidemiologic studies, supporting its utility in evaluating fracture risk at the population level [39, 40]. Fourth, as this study included only Korean participants, the results may not be generalizable to Western populations. Lastly, covariates such as income level may have changed over the 16‐year follow‐up period, which could potentially influence their association with fracture risk. Moreover, unmeasured confounders, including diet, medication use and vitamin D status, were not available in our dataset. Although we adjusted for multiple confounders, the inherent residual bias in retrospective cohort studies cannot be entirely excluded.

Despite the negative impact of obesity on health, the relationship between obesity and fractures is often overlooked. This study included a large sample of community‐dwelling individuals who were followed up for 16 years. Our findings clearly demonstrate an increased risk of fractures in individuals with high WHtR, regardless of age, sex or BMI. Relying solely on BMI to assess fracture risk may be inadequate, as our results suggest that obesity is not a protective factor for fractures. This highlights the need for a more comprehensive evaluation of obesity, particularly through measures like WHtR, to better understand fracture risk in the obese population.

## Conflicts of Interest

The authors declare no conflicts of interest.

## Supporting information


**Table S1** Association between WHtR and incident overall fractures based on sex.
**Table S2.** Association between BMI, WC and incident overall fractures based on sex.
**Table S3.** Association between WHtR and incident overall fractures based on age.

**Figure S1** Incidence rate of overall fracture according to obesity group and WHtR groups.

**Figure S2** Risk of overall fracture according to obesity groups and WHtR tertile groups.

**Figure S3** Scatter plots showing the relationship between WHtR and SoS at the midshaft tibia.

## Data Availability

The data are available through the Korea Disease Control and Prevention Agency (KDCA) and can be accessed with permission from the KDCA (http://www.kdca.go.kr).
