# Supplementary material for: Association Between Waist‐to‐Height Ratio Estimated Fat Mass Categories and Incident Fractures
Source: J Cachexia Sarcopenia Muscle. 2025 Jun 5;16(3):e13834. doi: 10.1002/jcsm.13834 (PMC12138268; doi:10.1002/jcsm.13834)
Supplement: Supplementary file 1 — Table S1 Association between WHtR and incident overall fractures based on sex. Table S2. Association between BMI, WC and incident overall fractures based on sex. Table S3. Association between WHtR and incident overall fractures based on age. [file JCSM-16-e13834-s003.docx]

**Content list**

**Supplementary Table 1. Association between WHtR and incident overall fractures based on sex**

**Supplementary Table 2. Association between BMI, WC and incident overall fractures based on sex**

**Supplementary Table 3. Association between WHtR and incident overall fractures based on age**

**Supplementary Figure 1. Incidence rate of overall fracture according to obesity group and WHtR groups**

**Supplementary Figure 2. Risk of overall fracture according to obesity groups and WHtR tertile groups**

**Supplementary Figure 3. Scatter plots showing the relationship between WHtR and SoS at the midshaft tibia**

**Supplementary Table 1. Association between WHtR and incident overall fractures based on sex**

|  | **Events** | **Person-years** | **Incidence rate*** | **Model 1** | | **Model 2** | | **Model 3** | | **Model 4** | |
| --- | --- | --- | --- | --- | --- | --- | --- | --- | --- | --- | --- |
|  |  |  |  | **HR**  **(95% CI)** | ***P*-value** | **HR**  **(95% CI)** | ***P*-value** | **HR**  **(95% CI)** | ***P*-value** | **HR**  **(95% CI)** | ***P*-value** |
| **Women** | | |  |  |  |  |  |  |  |  |  |
| per 1 SD increase |  |  |  | 1.52  (1.40– 1.65) | <0.001 | 1.59  (1.36– 1.85) | <0.001 | 1.59  (1.36– 1.82) | <0.001 | 1.56  (1.33– 1.83) | <0.001 |
| Group 1 | 77 | 16152 | 4.77 | 1.00 |  | 1.00 |  | 1.00 |  | 1.00 |  |
| Group 2 | 163 | 15496 | 10.52 | 2.21  (1.69– 2.90) | <0.001 | 1.92  (1.43–2.58) | <0.001 | 1.92  (1.43–2.57) | <0.001 | 1.87  (1.39–2.52) | <0.001 |
| Group 3 | 240 | 15001 | 16.00 | 3.36  (2.60– 4.35) | <0.001 | 2.73  (1.94– 3.82) | <0.001 | 2.73 (1.94–3.82) | <0.001 | 2.64  (1.87–3.72) | <0.001 |
| **Men** | |  |  |  |  |  |  |  |  |  |  |
| per 1 SD increase |  |  |  | 1.08  (0.97– 1.2) | 0.146 | 1.564  (1.29–1.88) | <0.001 | 1.55  (1.28–1.87) | <0.001 | 1.49  (1.22–1.81) | <0.001 |
| Group 1 | 101 | 13377 | 7.55 | 1.00 |  | 1.00 |  | 1.00 |  | 1.00 |  |
| Group 2 | 106 | 13264 | 7.99 | 1.05  (0.80– 1.38) | 0.703 | 1.54  (1.13– 2.11) | 0.007 | 1.54  (1.12– 2.11) | 0.007 | 1.45  (1.05–1.99) | 0.024 |
| Group 3 | 129 | 13156 | 9.81 | 1.30  (1.00– 1.68) | 0.052 | 2.53  (1.69– 3.78) | <0.001 | 2.50  (1.67–3.75) | <0.001 | 2.31  (1.53–3.49) | <0.001 |

*1,000 person-years

Model 1: unadjusted

Model 2: adjusted for age and body mass index

Model 3: adjusted for model 2: alcohol consumption, smoking, income, hypertension, diabetes, and thyroid disease

Model 4: adjusted for Model 3, SoS at the midshaft tibia, physical activity, and CRP level

*Abbreviations:* WHtR, waist-to-height ratio; HR, hazard ratio; CI, confidence interval; SoS, speed of sound; CRP, C-reactive protein.

**Supplementary Table 2. Association between BMI, WC and incident overall fractures based on sex**

|  | **BMI** | | | | **WC** | | | |
| --- | --- | --- | --- | --- | --- | --- | --- | --- |
|  | **Unadjusted** | | **Adjusted** | | **Unadjusted** | | **Adjusted** | |
|  | **HR**  **(95% CI)** | ***P*-value** | **HR**  **(95% CI)** | ***P*-value** | **HR**  **(95% CI)** | ***P*-value** | **HR**  **(95% CI)** | ***P*-value** |
| **Women** |  |  |  |  |  |  |  |  |
| per 1 SD increase | 1.08 (0.99–1.18) | 0.085 | 1.04 (0.94–1.14) | 0.452 | 1.46 (1.34–1.59) | <0.001 | 1.21 (1.10–1.33) | <0.001 |
| Group 1 | 1.00 |  | 1.00 |  | 1.00 |  |  |  |
| Group 2 | 1.12 (0.90–1.40) | 0.311 | 1.09 (0.87–1.37) | 0.440 | 1.73 (1.34–2.24) | <0.001 | 1.29 (0.99–1.69) | 0.059 |
| Group 3 | 1.16 (0.93–1.45) | 0.187 | 1.03 (0.82–1.29) | 0.790 | 2.72 (2.14–3.46) | <0.001 | 1.66 (1.28– 2.15) | 0.001 |
| **Men** |  |  |  |  |  |  |  |  |
| per 1 SD increase | 0.83 (0.75–0.93) | 0.001 | 0.92 (0.82–1.03) | 0.154 | 1.03 (0.93–1.15) | 0.535 | 1.06 (0.95–1.18) | 0.268 |
| Group 1 | 1.00 |  | 1.00 |  | 1.00 |  | 1.00 |  |
| Group 2 | 0.85 (0.66–1.09) | 0.193 | 0.94 (0.73–1.22) | 0.649 | 1.03 (0.79–1.34) | 0.844 | 1.10 (0.84–1.44) | 0.510 |
| Group 3 | 0.61 (0.47–0.80) | <0.001 | 0.74 (0.56–0.98) | 0.038 | 1.15 (0.88–1.49) | 0.299 | 1.21 (0.92–1.57) | 0.167 |

*Adjusted for age, alcohol consumption, smoking, income, hypertension, diabetes, thyroid disease, SoS at the midshaft tibia, physical activity, and CRP level

*Abbreviations:* BMI, body mass index; WC, waist circumference; HR, hazard ratio; CI, confidence interval; SoS, speed of sound; CRP, C-reactive protein.

**Supplementary Table 3. Association between WHtR and incident overall fractures based on age**

|  | **Events** | **Person-years** | **Incidence rate*** | **Model 1** | | **Model 2** | | **Model 3** | | **Model 4** | |
| --- | --- | --- | --- | --- | --- | --- | --- | --- | --- | --- | --- |
|  |  |  |  | **HR**  **(95% CI)** | ***P*-value** | **HR**  **(95% CI)** | ***P*-value** | **HR**  **(95% CI)** | ***P*-value** | **HR**  **(95% CI)** | ***P*-value** |
| **Age < 50** | | |  |  |  |  |  |  |  |  |  |
| per 1 SD increase |  |  |  | 1.21 (1.08– 1.37) | 0.002 | 1.62 (1.33– 1.97) | <0.001 | 1.63 (1.34–1.98) | <0.001 | 1.59 (1.30–1.95) | <0.001 |
| Group 1 | 89 | 19393 | 4.59 | 1.00 |  | 1.00 |  | 1.00 |  | 1.00 |  |
| Group 2 | 84 | 14817 | 5.67 | 1.24 (0.92– 1.67) | 0.163 | 1.52 (1.07– 2.14) | 0.018 | 1.51 (1.07–2.14) | 0.019 | 1.46 (1.03–2.07) | 0.034 |
| Group 3 | 70 | 9626 | 7.27 | 1.58 (1.16– 2.17) | 0.004 | 2.32 (1.48– 3.62) | <0.001 | 2.33 (1.49–3.64) | <0.001 | 2.32 (1.48–3.66) | <0.001 |
| **Age ≥ 50** | |  |  |  |  |  |  |  |  |  |  |
| per 1 SD increase |  |  |  | 1.24 (1.15– 1.35) | <0.001 | 1.54 (1.33– 1.79) | <0.001 | 1.55 (1.33– 1.79) | <0.001 | 1.50 (1.29– 1.75) | <0.001 |
| Group 1 | 89 | 10136 | 8.78 | 1.00 |  | 1.00 |  | 1.00 |  | 1.00 |  |
| Group 2 | 185 | 13943 | 13.27 | 1.51 (1.17– 1.95) | 0.001 | 1.68 (1.28– 2.20) | <0.001 | 1.68 (1.28–2.20) | <0.001 | 1.62 (1.24–2.13) | <0.001 |
| Group 3 | 299 | 18534 | 16.14 | 1.84 (1.45– 2.33) | <0.001 | 2.40 (1.77– 3.26) | <0.001 | 2.41 (1.77–3.27) | <0.001 | 2.29 (1.68–3.12) | <0.001 |

*1,000 person-years

Model 1: unadjusted

Model 2: adjusted for age, sex and body mass index

Model 3: adjusted for model 2: alcohol consumption, smoking, income, hypertension, diabetes, and thyroid disease

Model 4: adjusted for Model 3, SoS at the midshaft tibia, physical activity, and CRP level

*Abbreviations:* WHtR, waist-to-height ratio; HR, hazard ratio; CI, confidence interval; SoS, speed of sound; CRP, C-reactive protein.

**Supplementary Figure 1. Incidence rate of overall fracture according to obesity group and WHtR groups**

**
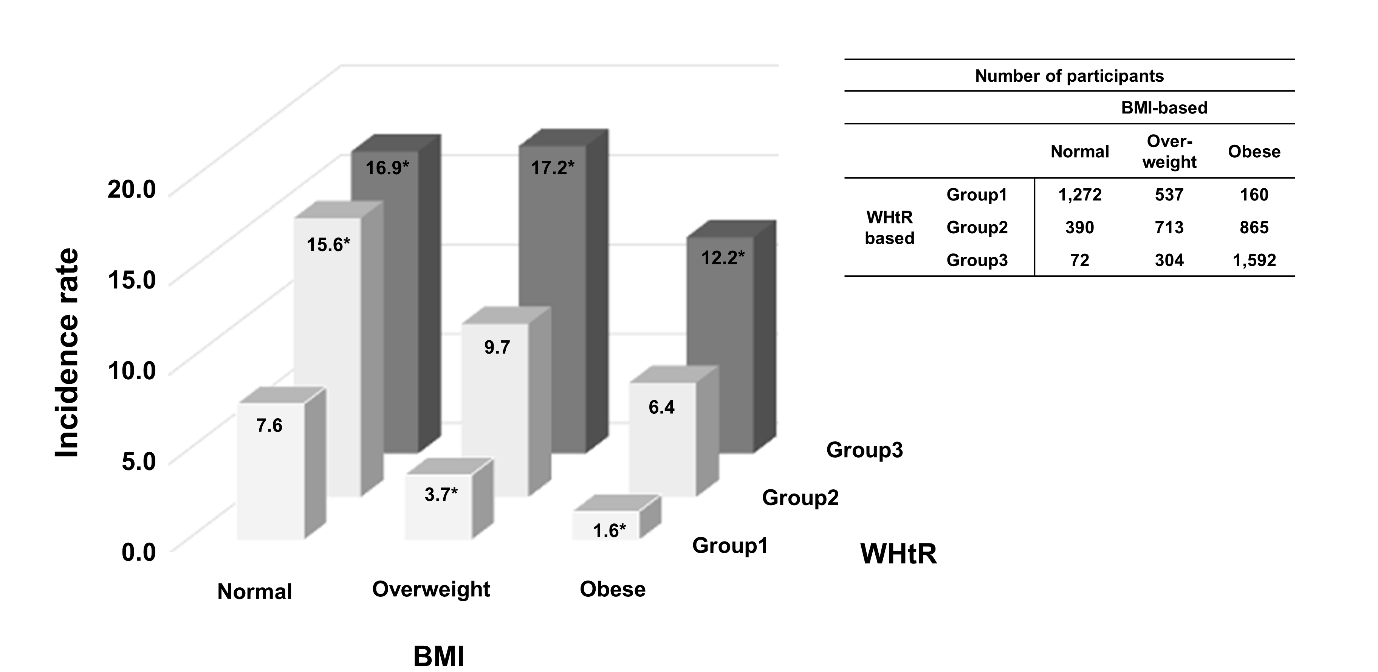
**

*Note:* Groups 1, 2, and 3 represent the WHtR tertile groups. Participants were also classified into three categories based on their BMI: obese (BMI ≥ 25 kg/m²), overweight (BMI 23–25 kg/m²), and normal weight. (BMI < 23 kg/m²). The incidence rate is presented in 1,000 person-years. The asterisk (*) indicates a significant difference compared to the reference group (normal weight and WHtR group 1) with a p-value < 0.05.

*Abbreviations:* BMI, body mass index; WHtR, waist-to-height ratio.

**Supplementary Figure 2. Risk of overall fracture according to obesity groups and WHtR tertile groups**


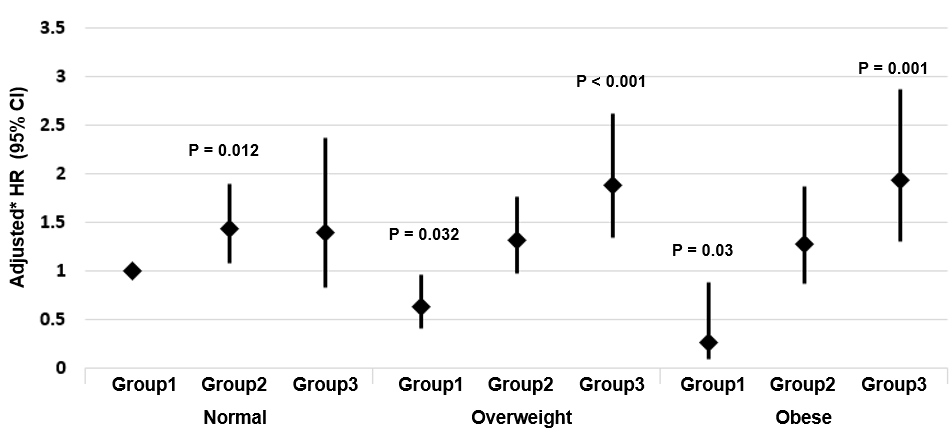


*Note:* Groups 1, 2, and 3 represent the WHtR tertile groups. Participants were also classified into three categories based on their BMI: obese (BMI ≥ 25 kg/m²), overweight (BMI 23–25 kg/m²), and normal/underweight (BMI < 23 kg/m²).

*Adjusted for age, sex, body mass index, alcohol consumption, smoking, income, hypertension, diabetes, thyroid disease, SoS at the midshaft tibia, physical activity, and CRP level

*Abbreviations:* WHtR, waist-to-height ratio; HR, hazard ratio; CI, confidence interval; SoS, speed of sound; CRP, C reactive protein.

**Supplementary Figure 3. Scatter plots showing the relationship between WHtR and SoS at the midshaft tibia**


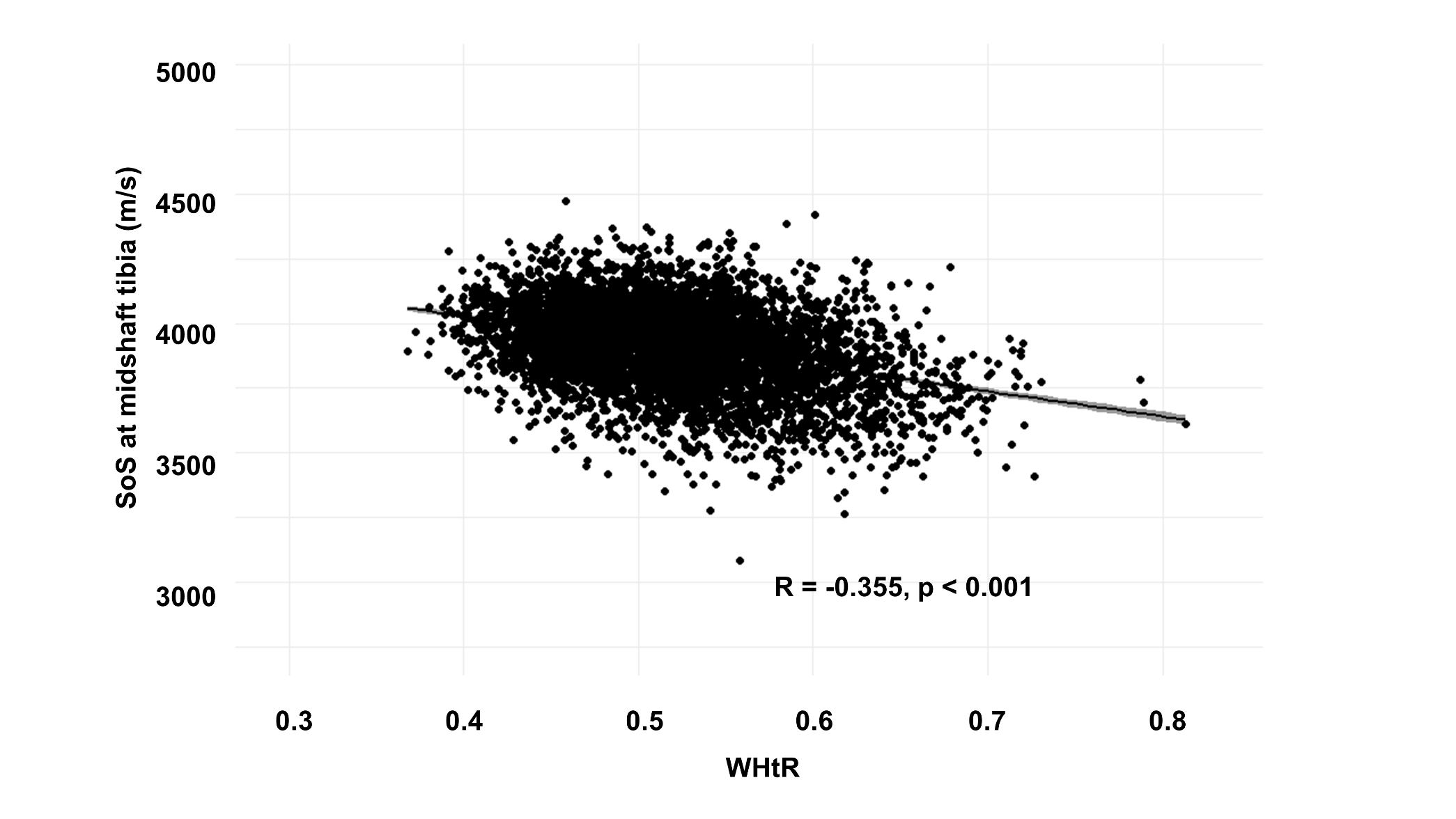


*Abbreviations:* WHtR, waist-to-height ratio; SoS, speed of sound.
