# Supplementary figures and images for: Association Between Waist‐to‐Height Ratio Estimated Fat Mass Categories and Incident Fractures
Source: J Cachexia Sarcopenia Muscle. 2025 Jun 5;16(3):e13834. doi: 10.1002/jcsm.13834 (PMC12138268; doi:10.1002/jcsm.13834)

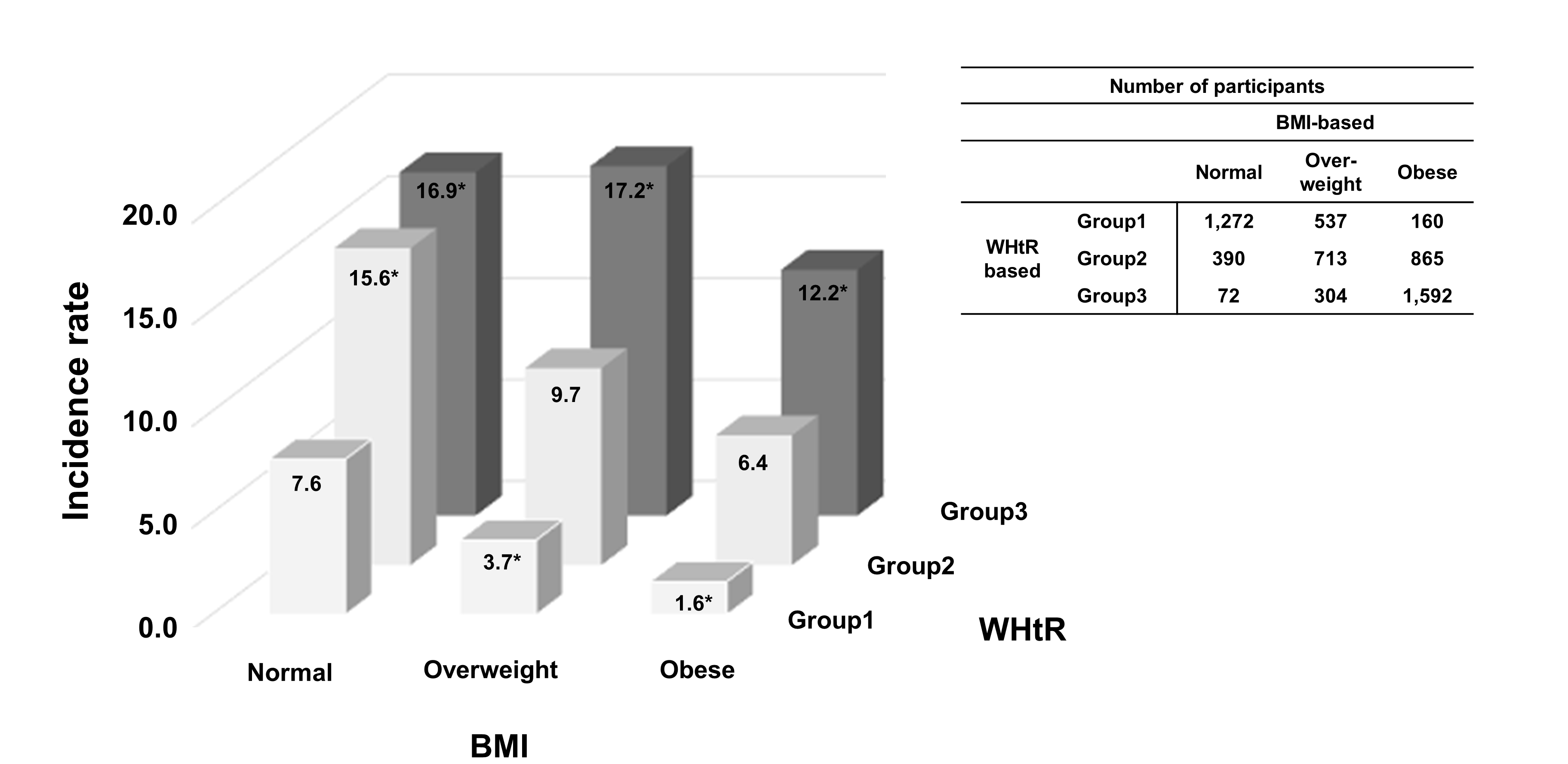

Supplement: Supplementary file 2 — Figure S1 Incidence rate of overall fracture according to obesity group and WHtR groups. [file JCSM-16-e13834-s002.tif]

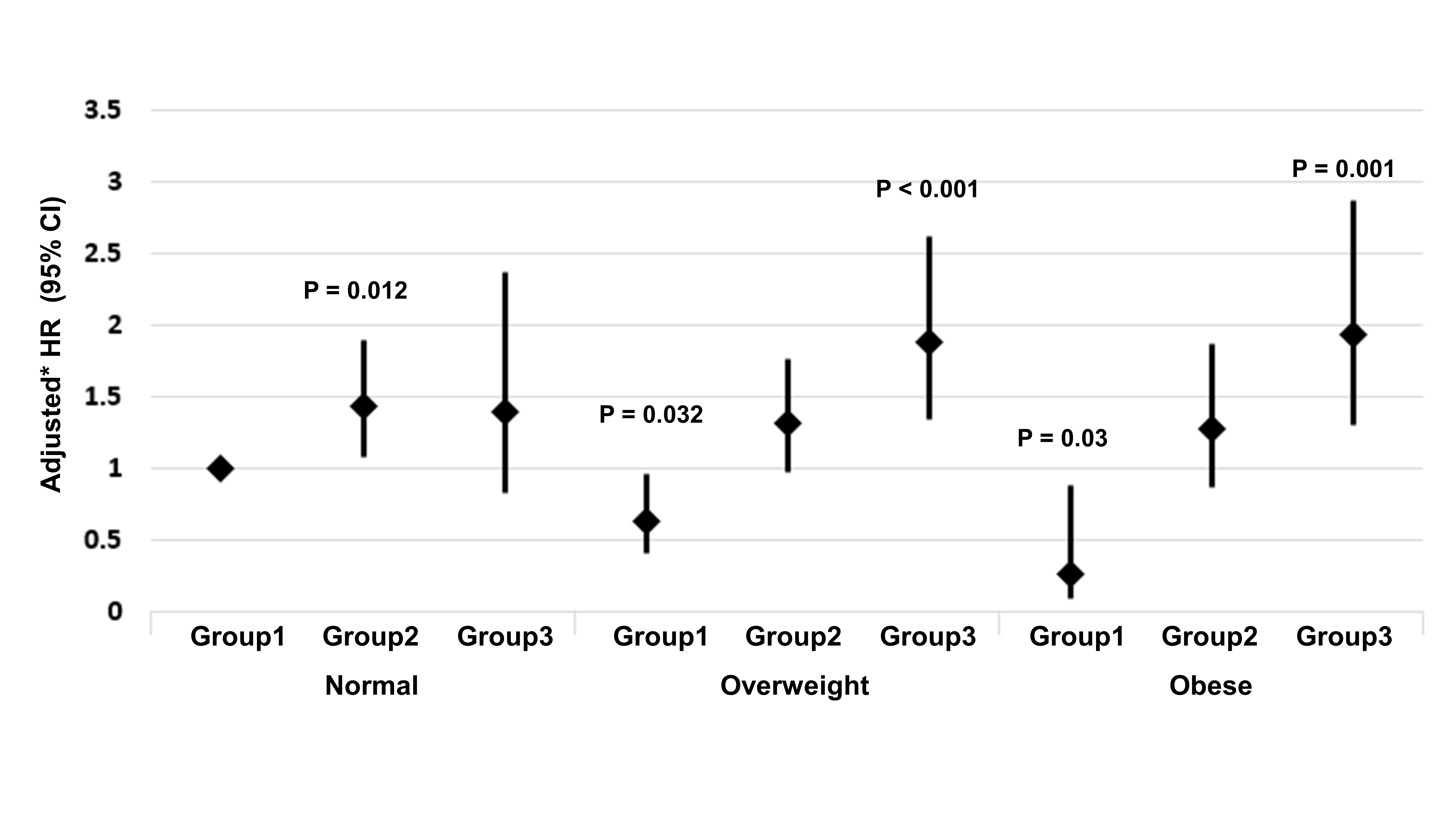

Supplement: Supplementary file 3 — Figure S2 Risk of overall fracture according to obesity groups and WHtR tertile groups. [file JCSM-16-e13834-s004.tif]

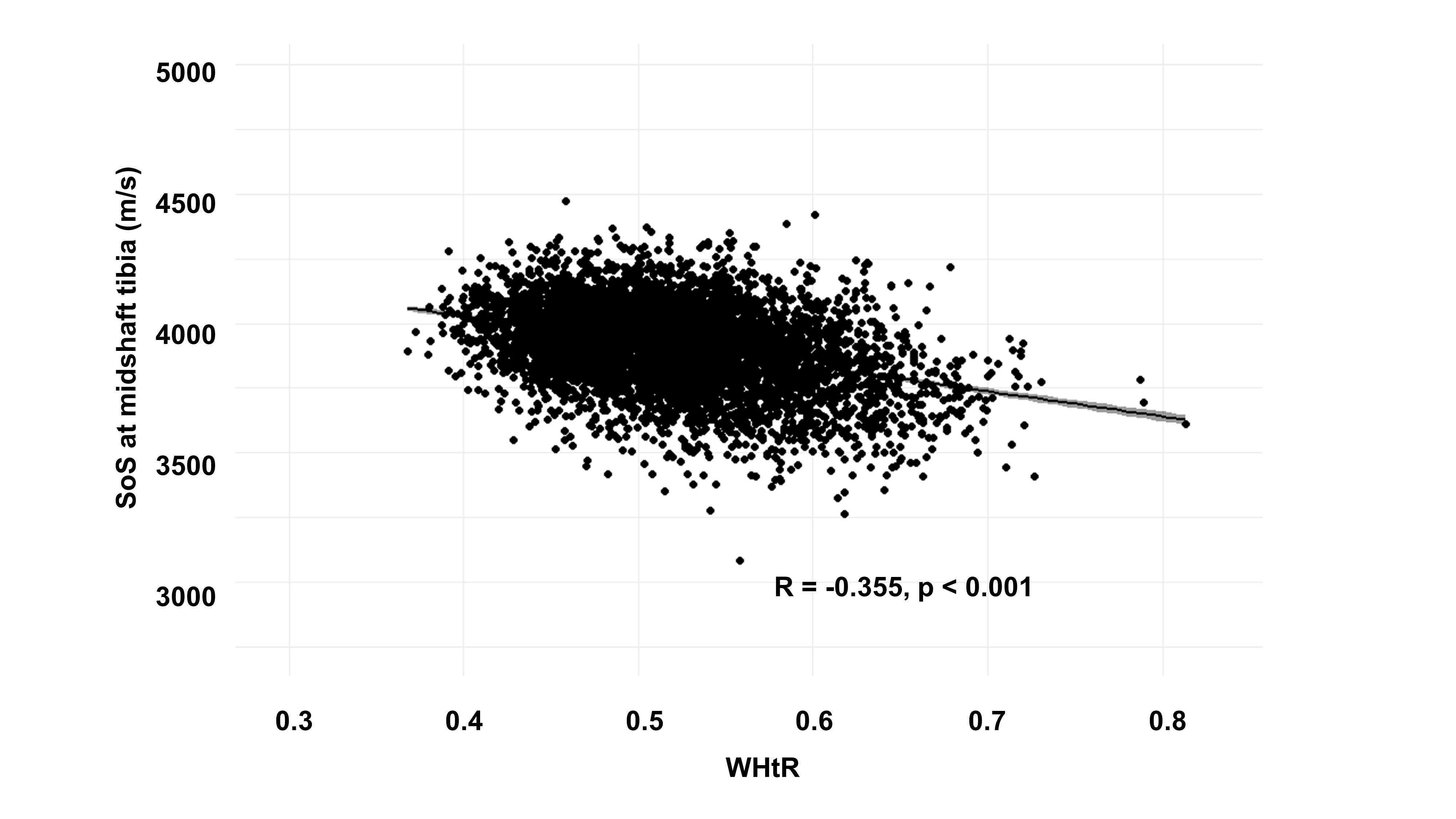

Supplement: Supplementary file 4 — Figure S3 Scatter plots showing the relationship between WHtR and SoS at the midshaft tibia. [file JCSM-16-e13834-s001.tif]
